# Predictors of Inpatient Mortality and Resource Utilization for the Elderly Patients With Chronic Hepatitis C (CH-C) in the United States

**DOI:** 10.1097/MD.0000000000002482

**Published:** 2016-01-22

**Authors:** Pegah Golabi, Munkhzul Otgonsuren, Winnie Suen, Aaron B. Koenig, Bashir Noor, Zobair M. Younossi

**Affiliations:** From the Betty and Guy Beatty Center for Integrated Research, Inova Health System, Falls Church, VA (PG, MO, WS, ABK, BN, ZMY); and Center for Liver Diseases, Department of Medicine, Inova Fair Falls Church, VA (WS, ZMY).

## Abstract

New incidents of chronic hepatitis C (CH-C) have stabilized yet the full impact of CH-C is not realized.

Assess inpatient mortality and resource utilization for CH-C patients hospitalized in the United States.

Adult CH-C patients were identified from The National Inpatient Sample (NIS) 2005 to 2009 database using the International Classification of Disease, Ninth Revision (ICD-9) diagnosis codes (070.51, 070.54, 070.70, 070.71, 070.41, and 070.44) also used to identify comorbidities.

324,823 hospitalized CH-C patients were identified. Of these, 13.63% (N = 44,288) were older than 65. The rate of hospitalization for the elderly cohort steadily increased over the study period with Medicare as the payer for the majority (86%). This cohort had higher inpatient charges, approximately a half day longer hospital stay (*P* < 0.001) and more moderate or severe illness. During the index hospitalization, older CH-C patients were twice more likely to die than the younger age-group (5% versus 2%, *P* < 0.001). In the adjusted model, older age (OR: 1.02 [95% CI, 1.02–1.03]), severity of illness (OR: 12.06 [95% CI, 10.68–13.62]), and number of diagnoses (OR: 1.10 [95% CI, 1.09–1.11]) were associated with higher in-hospital mortality; severity of illness and having private insurance were significantly associated with charge per hospital stay (*P* < 0.001).

The number of CH-C patients 65 and older increased due to the aging of the baby boomer population. Early treatment of CH-C patients with highly effective, well-tolerated, new anti-HCV regimens may prevent this significant societal burden.

## Abstract 2

Supplemental Digital Content is available in the text

## INTRODUCTION

Hepatitis C virus (HCV) infection is a major public health problem in the United States (US). HCV is the leading cause of cirrhosis and liver cancer and the most common reason for liver transplantation in the US.^1–7^ Despite the fact that the new cases of HCV have been decreasing, nearly 3 million new HCV patients are detected worldwide each year with 25,000 new cases in US.^8^ It is estimated that hepatic decompensation and HCC are expected to double by 2020 and by the same time liver-related deaths are expected to triple.^5^ The Center for Disease Control and Prevention (CDC) estimates that 3.2 million people have been exposed to HCV in the US and ∼75% to 85% of these patients have active viremia.^1^ The progression of chronic HCV infection typically occurs >2 to 3 decades and can be accelerated by alcohol consumption, and other host factors such as type 2 diabetes.^9^ In addition to mortality, patients with HCV also suffer from extrahepatic manifestations, chronic fatigue, arthralgias, emotional depression, and reduced physical and social functioning.^10–12^

A generation that has an increased risk for the presence of HCV is the Baby Boomers (Americans born between 1945 and 1965). Despite the high prevalence of HCV in this population, most are unaware of their HCV status allowing their chronic liver disease to mature and present only when complications such as cirrhosis are present.^13,14^ Therefore, due to the relatively high prevalence of HCV in this cohort, the burden of the liver disease, and its complications, the CDC has recommended a 1-time HCV screening for the Baby Boomer cohort.^15^ In addition, the increased life expectancy of the general population is also expected to contribute to an increase in HCV-related complications and for patients to seek care in the inpatient setting.^16^ Therefore, the aim of this current study was to assess inpatient mortality and resource utilization among older hospitalized patients with HCV diagnosis in the United States as compared to younger patients.

## METHODS

### Design

The National Inpatient Sample (NIS) is a retrospective, cross-sectional study that uses a 20% stratified random sample from all discharge from community hospitals (excludes rehabilitation and long-term acute care hospitals) to represent >95% of the U.S. population. The NIS database is maintained by the Agency for Healthcare Research and Quality and contains ∼8 million hospital discharges from about >1000 hospitals.

In this study NIS data was used and the study was approved by the Institutional Review Board of Inova Fairfax Hospital.

### Patient Population

From all adult patients (20 years or older) hospitalized between 2005 and 2009 (n = 32,697,993), we selected patients with hepatitis C virus (HCV) diagnosis [n = 500,617 (1.5%)] using International Classification of Disease, Ninth Revision (ICD-9) codes related to HCV (070.51, 070.54, and 070.70). We excluded patients co-infected with hepatitis B (n = 23,372), HIV (n = 21,447), superimposed alcoholic liver disease (n = 130,579), and those with missing information on age (n = 396). The final analytical cohort was 324,823.

### Outcome Variables

The primary outcome variable was the in-hospital mortality. Secondary outcome variables included inpatient LOS and total inpatients charges.

### Study Variables

#### Sociohemographics and Clinical Variables

Age (years), race (White, Black, Hispanic, Others), gender, median household income national quartiles for patient's ZIP Code, admission type (elective/trauma/other, ER/urgent), discharge status (routine, transfer to short-term hospital/other transfers including skilled nursing facility/intermediate care/another type of facility/home health care, against medical advice), severity of illness (minor/moderate, major/extreme loss of function), insurance type (Medicare, Medicaid, private, un-insured), number of diagnoses, and number of procedures were available.

#### Health Insurance

Medicare is a US government sponsored health insurance program for US residents aged 65 and older, for younger patients with disabilities and those with end-stage renal disease (ESRD) or amyotrophic lateral sclerosis.^17^ Also, detailed information about Medicaid program can be found on their website.^18^

### Data Analysis

Descriptive statistics are reported as median with interquartile for numerical variables and as frequencies with percentages for categorical variables. Initially, simple comparisons of numerical variables by age-group (< 65 years old versus 65 +) examined with 2-sided *t* test and categorical variables with chi-square tests. Characteristics of sociodemographic and clinical data were also explored according to Baby-Boomer time period (Additional Materials Table 1).

The logistic regression model to estimate odds ratios (ORs) with 95% confidence intervals (CIs) was utilized to identify factors associated with in-hospital mortality. Further, for our secondary outcome variables (LOS and charge), we calculated the betas with 95% CIs by simple linear regression models. We examined risk estimates of cost and LOS after exclusion extreme values due to a high left skewed data, but found similar associations in both models. Therefore, data are shown without exclusions of extreme values in the total sample. Data were examined stratified by the age group (20–64 years old and 65 or more) due to confounding from age. However, in-hospital mortality ORs estimates are presented for the age group (64 or less versus 65 or more) combined because stratification by the age group did not show substantial different results. Moreover, for final multivariate adjusted models of hospital charge and LOS, due to multi-collinearity among age, number of diagnoses, severity of illness, and expected primary payer, we did not adjust for age and number of diagnoses for hospital charge outcome model in younger group cohort, and we also did not adjust for number of diagnoses and expected primary payer (in the younger age group cohort) for LOS outcome).

## RESULTS

### Sociodemographic Data

From a total of 500,139 claims, 324,823 records remained for the final analytic cohort after the exclusion criteria (Table 1). 280,535 patients composed the younger group (patients < 65 years of age) whereas 44,288 patients composed the older group (65 years of age or older). A total of 208,810 patients in the younger group (74.4%) were among the Baby Boomer cohort. Forty-two percent of the younger group and 52% of the older patients were women (*P* < 0.0001). In the younger group, 59.7% of the patients were White and 22.6% of the subjects were Black, whereas these ratios were 56.6% and 18.8%, respectively, in the older group (*P* < 0.0001). Mean length of stay was 3.1 days for the younger group and 3.8 days for the older patients (*P* < 0.0001). The number of patients admitted to the hospital showed an increase during the study years, from 47,913 to 62,464 for the younger group and from 8,017 to 9,857 for the elderly group.

**TABLE 1:**
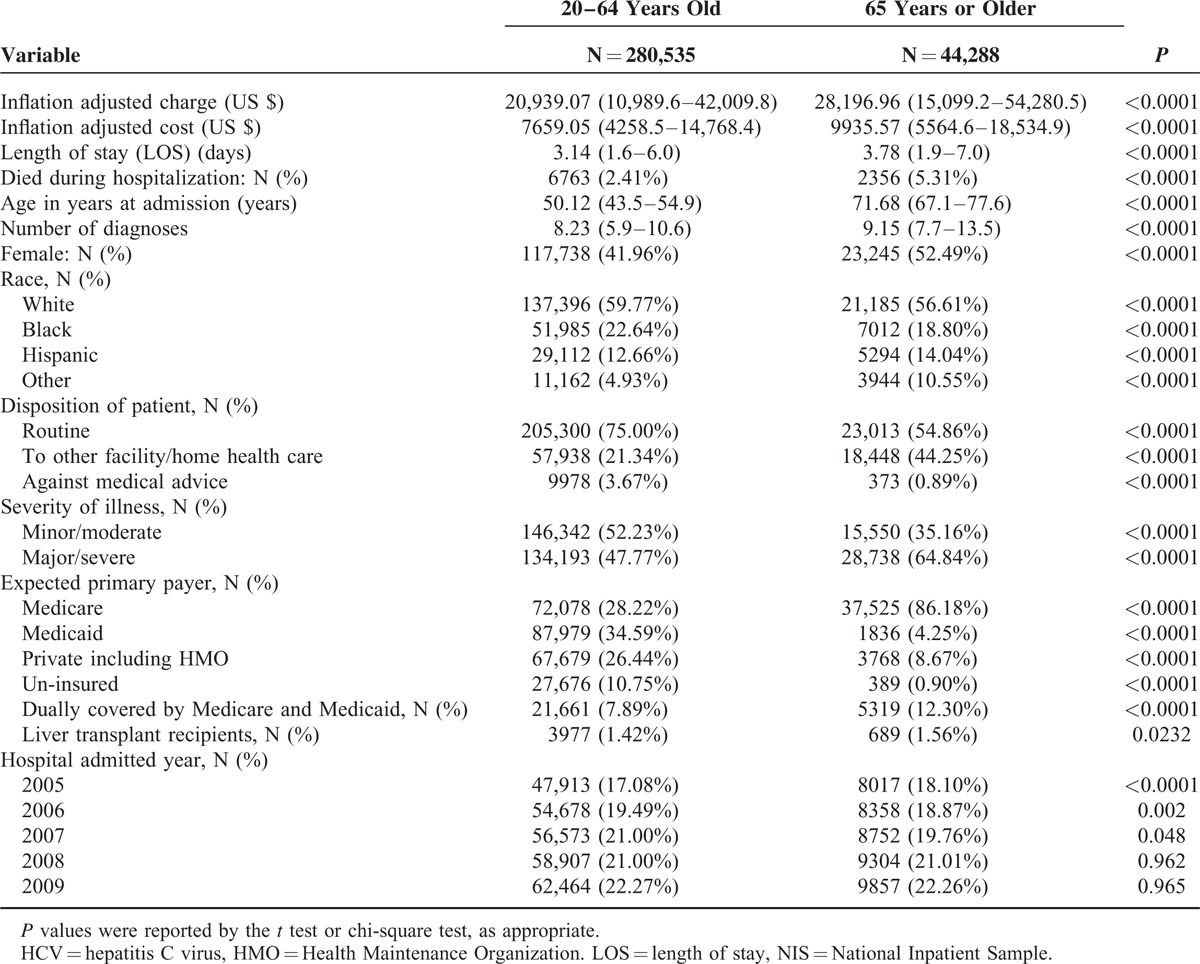
Study Characteristics of Patients With HCV, by Age Group, NIS, 2005 to 2009

### Predictors of Mortality

In-hospital mortality was 2.4% in the younger group and 5.3% among the older patients (*P* < 0.0001). Whereas major/severe illness was present in 65% of the older patients, it was present in 48% in the younger group (*P* < 0.0001). Mean number of diagnoses was 8.2 for the younger patients and 9.1 for the older group (*P* < 0.0001) (Table 1).

In multivariate analysis in the entire cohort, the presence of major/severe illness (OR:12.06 [95% CI: 10.68–13.62]) and having higher number of diagnoses (OR:1.10 [95% CI: 1.09–1.11]) were associated with increased risk of in-hospital mortality, whereas being female (OR: 0.80 [95% CI: 0.76–0.84]) and being admitted in the recent years (OR:0.55 [95% CI: 0.48–0.63]) were associated with decreased risk of in-hospital mortality (Table 2).

**TABLE 2:**
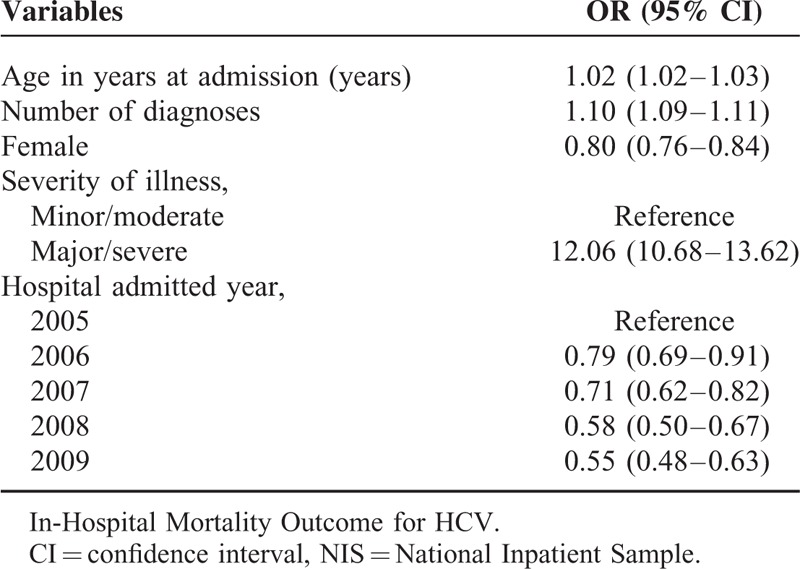
Multivariate-Adjusted Logistic Model on In-Hospital Mortality, NIS, 2005 to 2009

### Predictors of Resource Utilization

After inflation adjustment, mean hospital charge was $20,939 in the younger group and $28,196 in the older group. Also, again after inflation adjustment, mean cost was $7659 for the younger patients and $9935 for the older patients (all *P* < 0.0001). Medicare was the primary payer of 86% of patients in the older group and 28% of the younger group (*P* < 0.0001). Similarly, although 11% of the younger group was uninsured, this ratio was only 1% in the older group (*P* < 0.0001) (Table 1).

In multivariate analysis, in terms of inpatient charges, having a higher number of diagnoses and being hospitalized in recent years were both positively and significantly associated with higher inpatient charges whereas having Medicare coverage was associated with lower inpatient charges (Table 3).

**TABLE 3:**
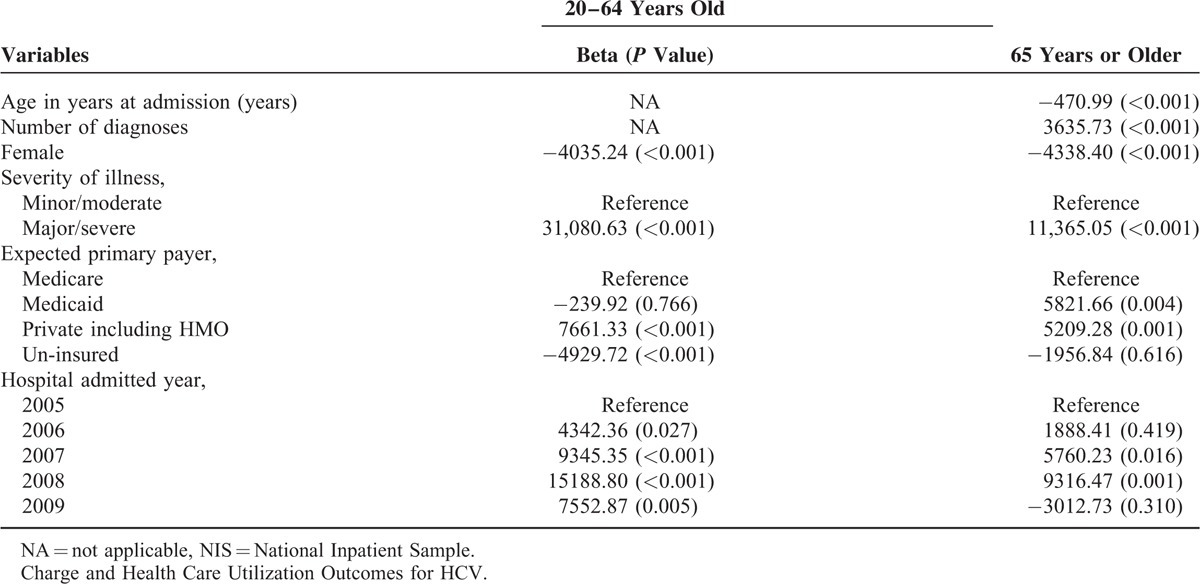
Multivariate-Adjusted Linear Regression Model on the Total Charge, by Age Group, NIS, 2005 to s2009

Also, being sicker (Beta = 3.6, *P* < .001) and having Medicaid as a primary payer (Beta = 1.2, *P* < .001) were positively associated with higher LOS (Table 4).

**TABLE 4:**
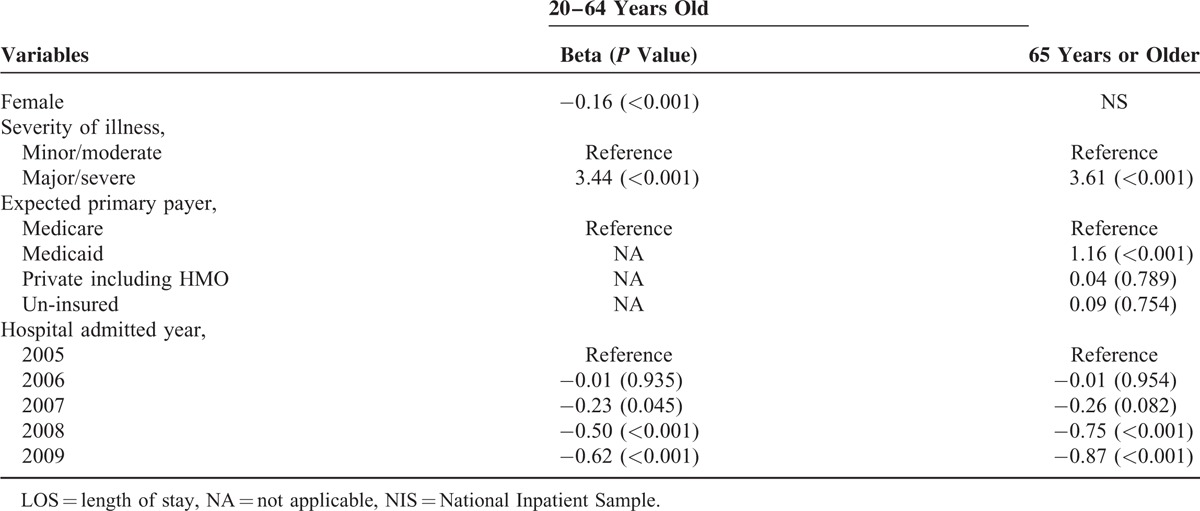
Multivariate-Adjusted Linear Regression Model on LOS, by Age Group, NIS, 2005 to 2009

## DISCUSSION

In this study we have shown that the number of HCV patients increased between 2005 and 2009, but in-hospital mortality decreased for all CH-C patients. Not surprisingly, the number of diagnoses and severity of illness significantly increased in-hospital mortality. Also, along with higher number of diagnoses, being admitted in the recent years was associated with increased inpatient charges. Our findings are in parallel with previous studies that stated the increasing pattern of the number of patients and resource utilization, as well as decreasing inpatient mortality.^19–21^

An important finding of our study was that, in the adjusted model, older age, severity of illness, and number of diagnoses was associated with higher in-hospital mortality. Interestingly, although the number of chronic HCV patients >65 years of age requiring hospitalization steadily increased over the study period, in hospital mortality decreased from 2005 to 2009. This finding was supported by a study of Luo et al, in which the in-hospital mortality decreased from 8.2% to 6.4%.^22^ Also, in another study, the case fatality rate was found to decrease from 8.9 in 1998 to 7.9 in 2003 with a yearly relative decrease rate of 3%.^23^ The reason of this decrease may be the change in the setting in which CH-C patients sought care. It was shown that there is a shift from inpatient to outpatient setting in the elderly patients, which can explain the decrease in in-hospital mortality.^24^ Our study also revealed that for the elderly group being sicker and having Medicaid as a primary payer were positively associated with higher length of stay which in turn increased inpatient charges and resource utilization. Elderly patients stayed in the hospital significantly longer than the younger age group which is parallel to a study of Hernandez et al.^25^ This difference can be attributed to the severity of disease seen in elderly.

Our study also revealed that in patients >65 years of age, severity of illness, and having a private insurance including HMO as a primary payer were independently and positively associated with charge per hospital stay. This finding validates a previous study in which total costs of HCV-associated hospitalizations increased as a result of the increase in the number of hospitalized patients.^22^ Furthermore, it is known that patients with HCV infection tend to be heavy users of health care^24–26^ due not only to their HCV infection but associated extra hepatic manifestations such as immune-mediated kidney disease, vascular disease, and/or diabetes.^27^ More patients are living in the stage of cirrhosis and its subsequent complications, which we also found among the elderly patients in this study that may help to explain the increase in hospitalizations.^24,28^ Complications of advanced liver disease such as encephalopathy and some invasive procedures such as paracentesis, gastrointestinal endoscopy, and hemodialysis increase the cost of hospitalization.^29,30^ An effective outpatient follow-up program like the one developed by Morando et al (a hepatologist led special caregiving support team) can decrease the hospitalization rate and the total cost of the disease.^31^ Another effective strategy for reducing costs was discussed in a study by Manos et al, where they showed that individuals with sustained virologic response (SVR) after antiviral treatment had nearly 2.5 times lower hospitalization rate than patients without SVR.^32^

Another important but expected finding of our study revealed that Medicare was the payer for >86% of CH-C patients >65 years of age. As previously shown, although average total yearly payments for inpatient claims for patients with HCV remained stable (2005 and 2010), the total charges submitted to Medicare by hospitals increased significantly.^24^ This data is especially important regarding the fact that in 2009, the last year of our study period, the oldest members of the Baby Boomer cohort were at the age of 64 and people >65 years of age constituted 12.5% of the US population.^33^ It is projected that in 2030, when the youngest members of Baby Boomer cohort will be eligible for Medicare, people aged 65 and older will comprise 20% of the US population.^34^ Indeed, the Baby Boomer cohort, who comprised >74% of our younger age group (Additional Materials Table 1), is especially important because of the possible effects on healthcare usage. As the HCV Baby Boomer become older, their liver disease will become more advanced requiring hospitalization. Indeed, this change was shown in a recent study by Zalesak et al. They have found that although Baby Boomers accounted for 75% of all HCV cases in US, this cohort constituted 84% of all advanced liver disease cases.^35^ In this context, our data indicate that this trend can already be documented in the inpatient data collected from 2004 to 2009.

Despite witnessing these increases in hospitalization, these rates are expected to rise. In fact, data suggest that regardless of reported low prevalence of HCV among elderly population (∼1%),^34^ the prevalence of HCV among elderly patients residing in nursing homes was 4.5%.^36^ Therefore, over the next decade, there will be increasing number of elderly patients with HCV. As previously shown, this higher prevalence of HCV in this group will lead to the higher rate of hospitalization^37^ and complications of cirrhosis.^38^ The fact that our data showed higher rates of disease severity in this group of patients is even more worrisome. In fact, it was disease severity that independently contributed to increased mortality and resource utilization. These findings were consistent with those from Gramenzi et al, confirming that elderly CH-C patients with higher disease severity were at higher risk for complications.^39^ Again, this data support the notion that age and indirectly duration of infection were associated with advanced fibrosis which a surrogate for mortality.^40^

Our study also has some limitations. First of all, when choosing study population, we excluded patients co-infected with hepatitis B (n = 23,372) and HIV (n = 21,447) in an attempt to decrease selection bias. Indeed, co-infection with other agents may increase disease severity, which in turn may increase mortality and healthcare usage. Also, it was previously shown that there was a shift of patients from inpatient to outpatient setting, with a substantial increase in outpatient numbers and resource utilization.^17,41^ In the present study, we only focused on the inpatient data and could not assess the changes in the outpatient setting, which were likely to affect inpatient findings. Also, as our entire study cohort was composed of patients with CH-C, we could not make comparisons with matched controls for each group. However, this comparison can be analyzed in another study.

In summary, older patients with CH-C will become an increasingly larger group as baby boomers with a high prevalence of HCV age over the next decade. This cohort will significantly contribute to higher inpatient mortality and resource utilization. As treatment with highly effective and well-tolerated regimens for HCV is available, treating this group could potentially reduce the burden of mortality and resource utilization in United States.

## Supplementary Material

Supplemental Digital Content
