# Supplementary material for: Predictors of Inpatient Mortality and Resource Utilization for the Elderly Patients With Chronic Hepatitis C (CH-C) in the United States
Source: Medicine (Baltimore). 2016 Jan 22;95(3):e2482. doi: 10.1097/MD.0000000000002482 (PMC4998257; doi:10.1097/MD.0000000000002482)
Supplement: Supplemental Digital Content [file medi-95-e2482-s001.docx]

**Additional Materials**

**Table 1.** Study socio-demographic characteristics of patients with HCV, by baby-boom period (1946-1964) age group, NIS, 2005-2009

| **Variable** | **Older than baby-boomers**  **(64 years old or older)**  **N = 56,738** | **Baby-boomers**  **(41 -63)**  **N = 208,810** | **Younger than baby-boomers**  **(20 - 40)**  **N = 59,275** | **P value *** | **P value** † |
| --- | --- | --- | --- | --- | --- |
| **Inflation adjusted charge (US $)** | 27625.80  (14693.6 - 53390.8) | 22546.79  (11893.8 - 44704.5) | 15372.02  (8443.0 - 30591.5) | <.0001 | <.0001 |
| **Inflation adjusted cost (US $)** | 9798.89  (5454.0 - 18427.5) | 8161.36  (4550.8 - 15669.5) | 5887.83  (3399.7 - 11032.1) | <.0001 | <.0001 |
| **Length of stay (LOS) (days)** | 3.73 (1.9 - 6.9) | 3.21 (1.6 - 6.1) | 2.86 (1.5 - 5.4) | <.0001 | <.0001 |
| **Died during hospitalization:** N (%) | 2893 (5.10%) | 5676 (2.72%) | 550 (0.93%) | <.0001 | <.0001 |
| **Age in years at admission (years)** | 69.02 (64.5-76.0) | 51.67 (47.7-55.4) | 34.74 (29.0-39.2) | <.0001 | <.0001 |
| **Number of diagnoses** | 8.94 (7.6 - 13.1) | 8.37 (6.1 - 11.1) | 7.22 (5.0 - 9.0) | <.0001 | <.0001 |
| **Female:** N (%) | 28517 (50.26%) | 80795 (38.68%) | 31671 (53.40%) | <.0001 | <.0001 |
| **Race,** N (%) |  |  |  |  |  |
| White | 26617 (55.85%) | 100243 (58.47%) | 31721 (65.77%) | 0.1057 | <.0001 |
| Black | 9742 (20.48%) | 42697 (24.99%) | 6558 (13.53%) | <.0001 | <.0001 |
| Hispanic | 6714 (13.99%) | 20392 (11.88%) | 7300 (15.16%) | <.0001 | <.0001 |
| Other | 4603 (9.68%) | 7866 (4.66%) | 2637 (5.55%) | <.0001 | 0.004 |
| **Disposition of patient,** N (%) |  |  |  |  |  |
| Routine | 30963 (57.46%) | 150129 (73.89%) | 47221 (80.48%) | <.0001 | <.0001 |
| To other facility/home health care | 22200 (41.48%) | 46471 (23.08%) | 7715 (13.27%) | <.0001 | <.0001 |
| Against medical advice | 567 (1.06%) | 6122 (3.03%) | 3662 (6.26%) | <.0001 | <.0001 |
| **Severity of illness,** N (%) |  |  |  |  |  |
| Minor/moderate | 20667 (36.46%) | 102766 (49.25%) | 38459 (64.98%) | <.0001 | <.0001 |
| Major/severe | 36071 (63.54%) | 106044 (50.75%) | 20816 (35.02%) | <.0001 | <.0001 |
| **Expected primary payer,** N (%) |  |  |  |  |  |
| Medicare | 42065 (76.33%) | 58394 (30.64%) | 9144 (17.25%) | <.0001 | <.0001 |
| Medicaid | 4888 (8.86%) | 60302 (31.79%) | 24625 (46.39%) | <.0001 | <.0001 |
| Private including HMO | 7243 (13.14%) | 54829 (28.70%) | 9375 (17.60%) | <.0001 | <.0001 |
| Un-insured | 913 (1.66%) | 17050 (8.88%) | 10102 (18.76%) | <.0001 | <.0001 |
| **Dually covered by Medicare and Medicaid, N (%)** | 6351 (11.45%) | 17023 (8.33%) | 3606 (6.24%) | <.0001 | <.0001 |
| **Liver transplant recipients, N (%)** | 158 (0.27%) | 3505 (1.68%) | 1008 (1.77%) | <.0001 | 0.1146 |
| **Hospital admitted year,** N (%) |  |  |  |  |  |
| 2005 | 11630 (20.38%) | 35453 (16.88%) | 8847 (14.85%) | <.0001 | <.0001 |
| 2006 | 11714 (20.29%) | 40593 (19.20%) | 10729 (17.95%) | 0.0493 | 0.0203 |
| 2007 | 11644 (20.63%) | 41512 (19.91%) | 12169 (20.57%) | 0.1683 | 0.3238 |
| 2008 | 10933 (19.11%) | 44170 (20.96%) | 13108 (21.76%) | 0.0020 | 0.0250 |
| 2009 | 10817 (19.58%) | 47082 (23.05%) | 14422 (24.87%) | <.0001 | 0.0150 |

* P values were reported by t-test or chi-square test when baby-boomers group compared to older than baby-boomers group;

† P values were compared baby-boomers group to younger than baby-boomers group
